# Disease Salience Effects on Desire for Affiliation With In-Group and Out-Group Members: Cognitive and Affective Mediators

**DOI:** 10.1177/1474704920930700

**Published:** 2020-07-09

**Authors:** Murray Millar, Andrea Fink-Armold, Aileen Lovitt

**Affiliations:** 1 University of Nevada, Las Vegas, NV, USA

**Keywords:** disease threat, prejudice, affiliation, out-groups, in-group

## Abstract

This study tested the hypothesis that threats related to infectious diseases would make persons less willing to affiliate with out-groups and that feelings of disgust and beliefs about the out-group members would mediate this effect. To test this hypothesis, American participants of European descent were presented with either a disease threat or control threat. Then they were shown a photograph of someone of the same race or different race. Participants were asked to indicate whether they would avoid the target person and to state their emotional and cognitive responses to the person. As predicted, disease salience decreased the desire to affiliate with out-group members, and both feelings of disgust and beliefs about the infection risk posed by the target person mediated this relationship.

Evolutionary models have long recognized that behavioral, cognitive, and emotional reactions should depend on the salience of particular goals or motives present in different contexts (e.g., Buss & Schmitt, 1993; Gangestad & Simpson, 2000). Schaller and his colleagues have suggested that persons possess a set of psychological mechanisms that motivate behaviors designed to limit exposure to potential sources of disease (Schaller & Duncan, 2007). When a disease threat is salience, it serves as an important contextual cue that engages these psychological mechanisms (Schaller & Park, 2011). Given that other persons are potential sources of pathogens, disease salience has implications for many social behaviors. For example, research has indicated that increases in infectious disease salience influence preferences for symmetrical faces (Young et al., 2011), preferences for novel sexual partners (Hill et al., 2015), and willingness to conform (Wu & Chang, 2012).

An important social impact of disease salience is on responses toward members of out-groups. A considerable body of research has indicated that when the threat of disease is salient, persons have a tendency to express more prejudicial attitudes about out-group members (e.g., Duncan & Schaller, 2009; Lund & Boggero, 2014; Park et al., 2007). Further, there is evidence that increases in disease salience can cause persons to avoid interactions with out-group members (e.g., Schaller & Neuberg, 2012) and engage in more overt discriminatory behavior (e.g., Laakasuo et al., 2018). These researchers have argued that prejudicial and avoidance responses to out-group members may have been adaptive in our ancestral past because out-group members may have been a particular disease threat. That is, out-group members could have carried novel pathogens to which persons have less physical immunity, and out-group members may not have adhered to local norms regarding hygiene that restrict disease contagion (Murray & Schaller, 2016). Additionally, there is evidence that sufficiently different out-groups may activate avoidance responses similar to the responses activated by disfigured persons (Ackerman et al., 2009).

Evolutionary scholars exploring the relationship between disease threat and reactions to out-groups have primarily focused on the mediating role of the affective response of disgust. Their research has produced evidence that disease threats cause persons to react with disgust to out-group members in a manner similar to other potential sources of pathogens. The feelings of disgust appear to motivate the avoidance of out-group members (e.g., Vartanian et al., 2015).

Although past research has focused on feelings of disgust, it is also possible that disease threats may elicit cognitive responses about out-group members who are partially responsible for the increases in prejudice and avoidance. Disease threats may trigger the stereotypes associated with out-groups that produce feelings of disgust or perhaps disgust primes those stereotypic beliefs and makes them more available. It seems possible that beliefs about out-group members may reflect the putative causes suggested by Murray and Schaller (2016) for the development of the relationship between disease threat and out-group prejudice. That is, persons may believe that out-group members represent a particular infection risk because they carry novel diseases and fail to adhere to hygienic norms. If persons hold these beliefs, then it seems probable that these beliefs play a role in promoting prejudice and discrimination toward out-group members.

## Current Research

The purpose of the present study was to explore the mediating role of these beliefs. Essentially, are these beliefs (anomalousness appearance, infection risk, violation of disease-prevention norms) part of the reason that persons have a desire to avoid out-group members when a disease threat is salient? To address this question, participants were presented with either a disease threat or control threat. Then, they were shown a photograph depicting someone of the same race or different race. While viewing the photograph, participants were asked to give their initial impressions of the person in the photograph. They were asked to indicate how likely they would be to avoid the person and to indicate the infection risk posed by the person, how anomalous the person appeared, how likely the person would violate disease-reducing norms, and feelings of disgust associated with the person. It was predicted that when a disease threat was salient, participants would express a greater desire to avoid out-group members than in-group members. Further, it was expected that the same pattern of results would be obtained with each of the potential mediators. That is, disease salience should create stronger feelings of disgust, more concerns about infection risk, more concern about health norm violations, and stronger judgments about the anomalous appearance of out-group members than in-group members. In addition, it was predicted that beliefs (anomalousness, infection risk, violation of disease-prevention norms) and feelings of disgust would partially mediate the relationship between the manipulations (control vs. disease threat and in-group vs. out-group membership) and the desire to avoid out-group members.

## Method

### Participants

A power analysis using G*Power (version 3.1) indicated that a sample of at least 128 persons would be needed to have at least an 80% probability of detecting a medium-sized true effect in a two-factor analysis of variance (ANOVA; Faul et al., 2009). A sample more than twice this size consisting of 271 participants (139 women and 132 men) of European descent was recruited from the general community and a large university in the Southwestern United States. The study employed an electronic sign up procedure to recruit participants and participation in the study was limited to persons indicating that they were 18 years of age or older. The average age of the participants was 36, and the range of ages was 18–78 years of age. Participants were randomly assigned to view a photograph depicting someone of the same race or different race and to either the disease or accident threat salience conditions. Seven participants failed to properly complete the experimental protocol.

### Materials

Six photographs were used in the study that depicted a male’s head and shoulders with a whited-out background. Three of the photographs were of persons of African descent and three were of persons of European descent. In a pretest, each person in these photographs was rated by 17 participants on 7-point scale with end points of 1 (*physically unattractive*) and 7 (*physically attractive*). Pictures depicting persons rated a slightly above average in physical attractiveness (*M* = 5.04) were chosen for the current study.

### Procedure

At the beginning of the study, the participants were informed that the purpose of the study was to investigate how people with different personalities evaluated other persons. The participants were reassured that all of their responses would be completely confidential. Following the introduction, they completed a short demographic questionnaire that asked participants to indicate their sex, age, general state of health, and ethnicity.

#### Manipulation of disease and accident threats salience

Following the demographic questionnaire, participants were asked to carefully read a short paragraph that they would be tested on later in the study. Approximately, half the participants were randomly assigned to read a paragraph that presented information related to everyone’s vulnerability to infectious diseases.

Specifically, the paragraph presented information about influenza indicating that persons of any age can contract the illness and that it can lead to serious complications. After reading the paragraph, participants were asked to recall the last time they had encountered someone with the flu and to answer four questions about symptoms experienced by this person. The other participants read a short paragraph that presented information about a nondisease health threat. The paragraph presented information about car accidents indicating that anyone can be involved in a car accident and that these accidents can lead to extensive injuries. After reading the paragraph, participants were asked to recall the last time they had encountered a car accident and to answer four questions about the accident (see Miller and Manner, 2012, for a similar procedure). In a pretest, 22 participants rated each paragraph on 7-point scale with end points of (*very anxious/not very anxious, threatened/not threatened*, and *very fearful/not very fearful*). No significant differences between the disease threat and the accident threat paragraphs were found, *F*(s) < 1.

#### In-group/out-group manipulation

Following the salience manipulation, the participants were presented with one of the six photographs. The participants were asked to briefly think about this person in the photograph and imagine what the person might be like. Approximately half the participants viewed a person from the same race (Whites viewing Whites) and half viewed a person from a different race (Whites viewing Blacks).

#### Measurement of reactions to target persons in photographs

After viewing the photograph, the participants were asked to make some judgments about the target person by indicating their agreement with a number of statements on scales with end points of 1 (*strongly disagree)* and 7 (*strongly agree*). Statements were presented that were related to each of the three mediating variables. Perceptions of anomalously were measured with statements such as this person looks a little odd and this person has a strange appearance. Infection risk was measured with statements such as this person might carry unusual diseases that I could catch and this person poses an infection risk. Violations of health norms were measured with statements such as this person might not regularly wash their hands and this person might not have had their immunizations. In addition, a measure of desire for affiliation and disgust with the target person was included. Desire for affiliation was measured by indicating agreement with statements such as I would keep away from this person and I would not be a friend to this person, and disgust with statements such as this person makes me feel disgusted. The order of the measures of the mediator variables, affiliation, and disgust was randomized for each participant.

In order to test the hypotheses, several ANOVAs were conducted to explore whether the manipulations (threat type and group membership) interacted to influence both avoidance and the mediating variables (disgust, infection risk, health norms, and anomalous appearance). Following this, a series of regressions were conducted to demonstrate mediation by showing that the removal of the variance associated with the mediators would weaken the relationship between the manipulated variables and avoidance.

## Results

**Figure 1.**
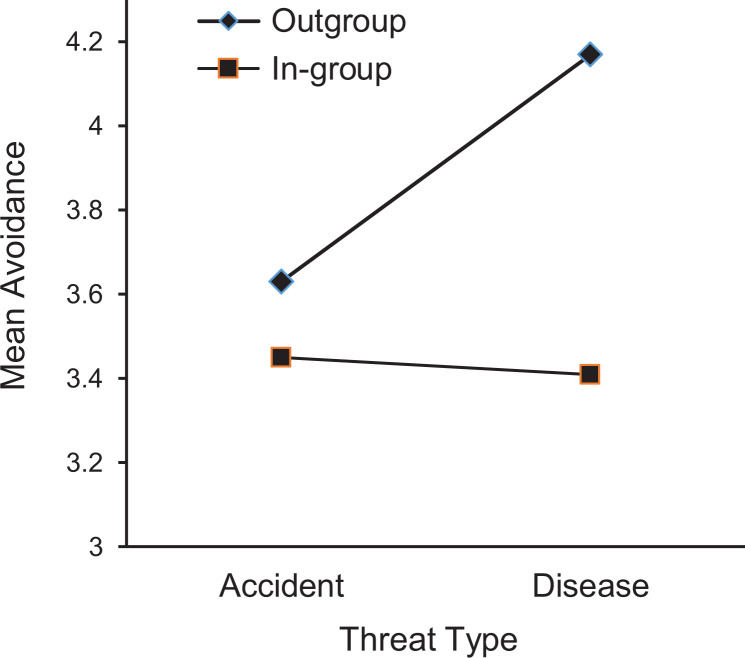
The mean avoidance scores as function of threat type and group membership. Higher scores indicate more avoidance.

The items used to measure each of the potential reactions to the person presented in the photographs were averaged for each participant to create a measure of avoidance (α = .95), disgust (α = .96), anomalous appearance (α = .91), infection risk (α = .94), and health norm violations (α = .87). To examine the hypothesis that disease threat would lead to more avoidance, more negative beliefs (infection risk, norms, and anomalous appearance) and more feelings of disgust each of these measures were analyzed in separate 2 (control vs. disease threat) × 2 (in-group vs. out-group membership) ANOVA. When avoidance was examined, a main effect for group membership was found with participants expressing a greater desire to avoid out-group members (African Americans; *M* = 3.88, *SD* = 1.20) than in-group members (White Americans; *M* = 3.34, *SD* = 1.16), *F*(1, 266) = 10.50, *p* = .001, $\eta_{\text{p}}^{2}$ = .04. This main effect was qualified by the expected threat type by group-membership interaction, *F*(1, 266) = 4.15, *p* = .04, $\eta_{\text{p}}^{2}$ = .02. When a disease threat was salient, participants expressed more desire to avoid out-group members than in-group members, *F*(1, 266) = 13.60, *p* < .001, $\eta_{\text{p}}^{2}$ = .05, and when a control threat was present, this difference disappeared, *F* < 1 (see Figure 1).

**Figure 2.**
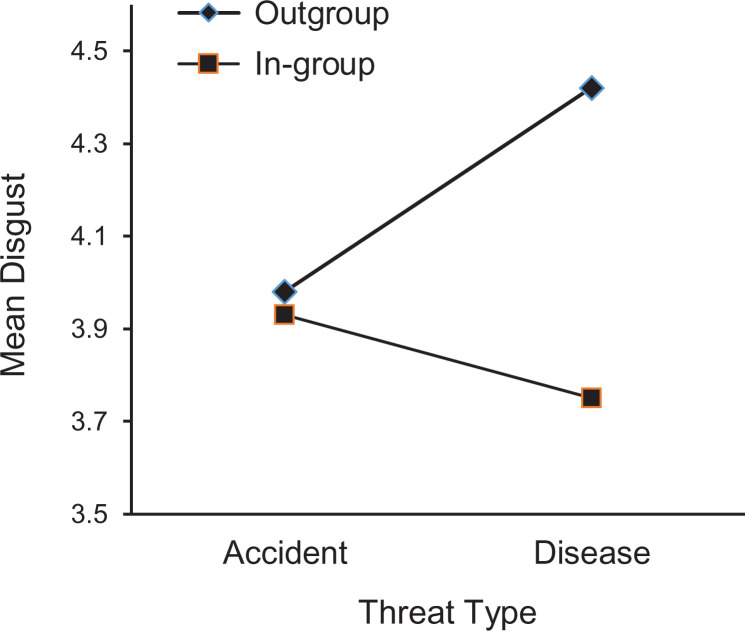
The mean disgust scores as function of threat type and group membership. Higher scores indicate more feelings disgust.

**Figure 3.**
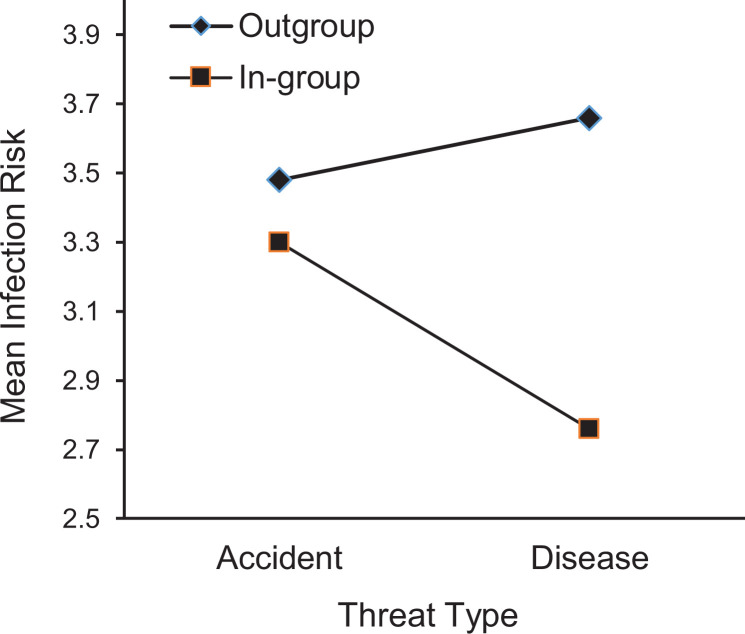
The mean infection risk scores as function of threat type and group membership. Higher scores indicate more perceived infection risk.

The same pattern of results was obtained when the potential mediators of disgust and infection risk were examined. Overall, participants indicated more feelings of disgust toward out-group members (*M* = 3.84, *SD* = 1.24) than in-group members (*M* = 4.19, *SD* = 1.22), *F*(1, 262) = 5.72, *p* = .02, $\eta_{\text{p}}^{2}$ = .02, and this was qualified by a threat type by group-membership interaction, *F*(1, 262) = 4.16, *p* = .04, $\eta_{\text{p}}^{2}$ = .02. If a disease threat was salient, participants expressed more disgust toward out-group members than in-group members, *F*(1, 262) = 14.33, *p* < .001, $\eta_{\text{p}}^{2}$ = .03, and if a control threat was present, this difference disappeared, *F* < 1 (see Figure 2). Similarly, participants indicated more concern about an infection risk from out-group members (*M* = 3.56, *SD* = 1.23) than in-group members (*M* = 3.03, *SD* = 1.24), *F*(1, 266) = 13.90, *p* < .001, $\eta_{\text{p}}^{2}$ = .05 and this was qualified by a threat type by group-membership interaction, *F*(1, 266) = 6.29, *p* = .01, $\eta_{\text{p}}^{2}$ = .02. In the disease threat conditions, infection risk was perceived as greater for out-group members than in-group members, *F*(1, 266) = 18.95, *p* < .001, $\eta_{\text{p}}^{2}$ = .067, and this was not found in the control threat conditions, *F* < 1 (see Figure 3).

The ANOVAs examining the health norms and anomalous appearance variables failed to find the predicted interaction between threat type and group membership. In both analyses, the only significant finding was a main effect for group membership. Participants believed that out-group members were more likely to violate health norms (*M* = 3.13, *SD* = 1.10) than in-group members (*M* = 2.84, *SD* = 1.10), *F*(1, 267) = 5.14, *p* = .02, $\eta_{\text{p}}^{2}$ = .02, and that out-group members (*M* = 4.05, *SD* = 1.15) had a more anomalous appearance than in-group members (*M* = 3.63, *SD* = 1.10), *F*(1, 267) = 10.00, *p* < .002, $\eta_{\text{p}}^{2}$ = .04.

### Mediational Analyses

A set of mediational analyses was performed to examine the hypothesis that feelings of disgust and beliefs would partially mediate the relationship between the interaction (Threat type × Group membership) and the desire to avoid out-group members. First, separate mediational analyses were conducted for each of the potential mediators (disgust and infection risk). These mediational analyses attempted to show that the influence of the interactive effect (Threat type × Group membership) on avoidance was mediated by changes in the mediator (disgust or infection risk). If disgust or infection risk mediates the impact of the interaction on avoidance, then the removal of the variance associated with the mediator should weaken this relationship and there should be a nonzero indirect effect of the interaction term through the mediator on avoidance.

To demonstrate this, separate two-step hierarchical regression analyses were performed for both of the potential mediators (disgust and infection risk). In the first step, avoidance was regressed on threat type, group membership, and the interaction term (Threat type × Group membership). The interaction term was created by centering the variables and multiplying the threat type variable by the group membership variable. In the second step, avoidance was regressed on the same variables in the first step (threat type, group membership, and the interaction term) with the addition of the mediating variable. To demonstrate the indirect effect of the interaction through the mediating variable a bootstrap procedure outlined by Hayes (2018) was used.

When disgust was examined, in the first step unsurprisingly in light of the ANOVA results, the interaction term significantly predicted avoidance, *b* = .61, *t* = 2.04, *p* = .02. In the second step, consistent with the mediational hypothesis, when the variance associated with disgust was controlled for by adding it to the model, the interaction between threat type and group membership was no longer a significant predictor of avoidance, *b* = .14, *t* = 0.72, *p* = .47. Further, consistent with the mediational hypothesis, there was evidence for an indirect effect of the interaction (Threat type × Group membership) through feelings of disgust on avoidance. The 95% confidence interval (CI) based on 5,000 bootstrap samples for the indirect effect (*b* = .45) did not contain a zero effect (CI [.02, .89]).

When beliefs about infection risk were examined, again in the first step, the interaction term significantly predicted avoidance, *b* = .69, *t* = 2.37, *p* = .02. In the second step, consistent with the mediational hypothesis, when the variance associated with infection risk was controlled for by adding it to the model, the interaction between threat type and group membership was no longer a significant predictor of avoidance, *b* = .27, *t* = 1.04, *p* = .30. In addition, there was evidence for the indirect effect of the interaction (Threat type × Group membership) through feelings of infection risk on avoidance (*b* = .32, 95% adjusted bootstrap with 5,000 samples CI [.06, .65]).

Having demonstrated that both disgust and beliefs about infection risk could act separately as mediators, another analysis was performed to demonstrate the combined mediational effects of both of these variables. A serial multiple mediational model was used in which the indirect effect of the interaction (Threat type × Group membership) on avoidance flows through disgust and then infection risk (interaction term > disgust > infection risk > avoidance). There was evidence for the combined mediating role of disgust and infection risk. The 95% CI based on 5,000 bootstrap samples for this indirect effect (*b* = .04) did not contain a zero effect (CI [.005, .12]).

### Control Analyses

It was important to examine whether the participants’ sex interacted with the manipulations because all the targets in the pictures were men. To examine this, the independent variables (avoidance, disgust, infection risk, anomalous appearance, and health norms) were analyzed in separate 2 (sex of the participant) × 2 (threat type) × 2 (group membership) ANOVAs. In all of these analyses, the sex of the participant did not interact with any other variable. The only significant effect found was when disgust was examined, overall, women reported more feelings of disgust (*M* = 4.22, *SD* = 1.17) than men (*M* = 3.83, *SD* = 1.28), *F*(1, 262) = 5.24, *p* = .02, $\eta_{\text{p}}^{2}$ = .02. Similarly, it is important to examine whether the age of the participant interacted with the manipulations. To examine this, a regression analysis was conducted in which age of the participant, threat type, in-group/out-group membership, and interactions of these variables were regressed on avoidance. The age of the participant was not involved in any significant effects. However, the distribution of ages in the sample did not allow for a particularly robust test of age effects.

## Discussion

The purpose of the current study was to investigate whether beliefs, in addition to feelings of disgust, mediated the relationship between disease salience and avoidance of out-group members. The findings provided partial support for the predictions. When a disease threat was salient, participants believed that out-group members posed a greater infection risk than in-group members. Further, these beliefs about infection risk mediated the relationship between disease threat and the desire to avoid out-groups. Finally, a multimediational model that included both feelings of disgust and beliefs about infection risk suggested that both the variables could play a simultaneous mediating role.

In addition, the results of the current study replicated a couple of significant findings found in the extant literature. First, the results add to the large corpus of research indicating that disease threats compared to other types of threats can motivate avoidance and prejudice toward members of the out-group (e.g., Schaller & Duncan, 2007; Schaller & Park, 2011). This is important because recently, some controversy about the interpretation of this relationship has arisen (Kusche & Barker, 2019). Second, the results provide another demonstration that disease threats are associated with more feelings of disgust toward out-group and that these feelings of disgust mediate the relationship between disease threat and avoidance of the out-group (e.g., Zakrzewska et al., 2019).

However, contrary to the predictions, the participants’ beliefs about violating health norms and anomalous appearance were uninfluenced by disease threat or group membership and did not act as mediators. The failure of beliefs about violating health norms is particularly puzzling in light of research indicating that persons do have negative beliefs about the health practices of out-group members (Priest et al., 2018). It is tempting to simply conclude that concerns about norm violations and anomalous appearance do not act as mediators. Yet it is also possible that the study’s procedures may have been responsible for the lack of findings. For example, the effects of anomalous appearance may have been obscured by utilizing photographs that were standardized across ethic groups in terms attractiveness, that is, in an effort to control attractiveness, all the persons in the photographs had all been rated as moderately attractive. Or perhaps the items used about health norm violations did not address the relevant health norms, that is, norms that the participants in this sample believed would be violated.

Beyond addressing the hypotheses, the results also produced a couple of other notable findings. First, the sample expressed relatively higher levels of prejudicial beliefs about the out-group (African Americans) than the in-group. This was true not only for infection risk and disgust but also for the variables not influenced by threat manipulation, that is, they perceived out-group members as more likely to violate health norms and more anomalous in appearance than in-group members. Unfortunately, this finding is consistent with a large literature that has examined the prevalence and promotion of stereotypic beliefs in the American population (e.g., Deskins et al., 2017) and some of these stereotypes include the endorsement of beliefs related to the relative health of African Americans (e.g., Priest et al., 2018). A second notable finding was, even though the sex of the participant did not interact with our manipulations (threat type and group membership), women overall reported more feelings of disgust at the thought of interacting with the targets than men. This finding is consistent with a large body of research and theorizing that has suggested women experience more disgust than men (e.g., Al-Shawaf et al., 2017).

### Issues and Limitations

First, it is important to recognize that there are host of other contextual factors beyond group membership that make individuals more or less responsive to the health threats posed by others. For example, an individual with poor health may be particularly concerned about the health threats posed by out-group members (Park et al., 2007). Also, during pregnancy, women often experience an increase disgust sensitivity that might increase their negative reactions to out-group members (Navarretea et al., 2007). The relationship between these other contextual factors and group membership will need to be explored.

Second, in the current study, out-group/in-group membership was operationalized by having European Americans evaluate either African or European American targets. It is possible that the current findings would not generalize to other non-African American out-groups. That is, European Americans may have a unique response to African Americans as opposed to other ethnic out-groups (e.g., Hispanics, Asian Americans). Further, it is conceivable that other non-European groups (e.g., Hispanics, Asian Americans) might respond differently when asked to evaluate out-group members. It is important to note that European Americans have been traditionally the majority and power-holding group in American society. There is the potential that minority groups may evaluate out-group members differently from the majority group. Future research should address both of these questions.

Finally, an issue in this study concerns how to explicate the relationship between the emotion of disgust and beliefs about infection risk. In the current study, a multimediational model was tested in which feelings of disgust lead to beliefs about infection risk. Of course lacking any meaningful temporal ordering of the measures the causal relationship between disgust and infection risk is ambiguous. It is plausible that the relationship could be reversed with thoughts about infection risk causing feelings of disgust or that disgust and beliefs about infection risk are causally unrelated and exert independent effects on the desire to affiliate. The relationship between and even the separation of emotional and cognitive responses has been controversial throughout the history of psychology (Lazarus, 1999), and more recent neuroscience data have suggested that emotion and cognition may be hopelessly and intricately entangled (Crocker et al., 2013). The current research did not attempt to address this issue but instead focused on the more limited goal of demonstrating that both disgust and the beliefs suggested by an evolutionary approach play a role in negative reactions to out-group members.
